# Study of radiation induced changes of phosphorus metabolism in mice by ^31^P NMR spectroscopy

**DOI:** 10.2478/v10019-010-0030-z

**Published:** 2010-09-09

**Authors:** Igor Sersa, Simona Kranjc, Gregor Sersa, Alenka Nemec-Svete, Bojan Lozar, Ana Sepe, Jernej Vidmar, Marjeta Sentjurc

**Affiliations:** 1 Jozef Stefan Institute, Ljubljana, Slovenia; 2 Department of Experimental Oncology, Institute of Oncology Ljubljana, Ljubljana, Slovenia; 3 Veterinary Faculty, Ljubljana, Slovenia

**Keywords:** X-ray irradiation, ^31^P NMR spectroscopy, creatine kinase, biological effects of radiation, radiation dosimetry

## Abstract

**Background:**

The aim of this study was to examine whether ^31^P NMR can efficiently detect X-ray radiation induced changes of energy metabolism in mice. Exposure to ionizing radiation causes changes in energy supply that are associated with the tissue damage because of oxidative stress and uncoupled oxidative phosphorylation. This has as a consequence decreased phosphocreatine to adenosine triphosphate ratio (Pcr/ATP) as well as increased creatine kinase (CK) and liver enzymes (transaminases AST and ALT) levels in serum.

**Materials and methods:**

In this study, experimental mice that received 7 Gy of X-ray radiation and a control group were studied by ^31^P NMR spectroscopy and biochemically by measuring CK and liver enzyme levels in plasma. Mice (irradiated and control) were measured at regular time intervals for the next three weeks after the exposure to radiation.

**Results:**

A significant change in the Pcr/ATP ratio, determined from corresponding peaks of ^31^P NMR spectra, was observed in the 7 Gy group 2 days or more after the irradiation, while no significant change in the Pcr/ATP ratio, was observed in the control group. This result was supported by parallel measurements of CK levels that were highly increased immediately after the irradiation which correlates with the observed decrease of the Pcr/ATP ratio and with it associated drop of muscle energy supply.

**Conclusions:**

The ^31^P NMR measurements of the Pcr/ATP ratio can in principle serve as an instantaneous and noninvasive index for assessment of the received dose of irradiation.

## Introduction

Biochemical changes in macromolecules and with that associated tissue damage appears several ms after acute exposure to radiation. However, multiple consequences manifest in hours, years or even decades after the irradiation. Since stochastic biological effects of radiation could be detected only through carefully planned epidemiological studies, several attempts have been made to develop a successful method for detecting deterministic effects of radiation, where the rate of tissue damage is proportional to a received radiation dose.^1^,^2^ Such methods may help determining received doses of radiation for all subjects that were at the time of exposure not equipped with radiation detection devices. This may have important applications in military use as well as in civil use: hospitals, nuclear power plants and in some industry branches.

The most promising are the methods based on the measurements of the long lived radiation induced stable radicals in the hydroxyapatite component of teeth and bones which can be measured by electron paramagnetic resonance (EPR).^3^ With the recent development of low-frequency EPR (1 GHz) the measurements *in vivo* on teeth seems to become plausible.^4^

The other challenge is to find the method by which it would be possible to measure direct biological effect that is proportional to the dose of radiation. According to Bergonié-Tribondeu’s law^5^, where the radiosensitivity of cells is proportional to their reproductive activity and inversely proportional to their differentiation level, only spermatogonia and erythroblasts are highly radiosensitive to radiation. Therefore, assessment of the radiation damage through methods, which detect the DNA damage^6^, *i.e.*, gene aberration detection methods^7^ or the FISH method^8^,^9^, is found to be rather complicated and time consuming due to a need of gathering specific samples. Effects of radiation are associated also with a skeletal damage, which can be detected by MR microscopy.^10^ Radiation has significant effect also on metabolism of living systems that is linked with changing concentrations of phosphocreatine (Pcr) and adenosine triphosphate (ATP) molecules. Pcr, also known as creatine phosphate, is an important molecule for energy storage in skeletal muscles. Pcr is used to generate ATP by transferring phosphate group to the adenosine diphosphate molecule (ADP) forming creatine for the 2 to 7 seconds following an intense anaerobic effort. ADP conversion to ATP occurs in a catalytic reaction catalyzed by creatine kinase (CK). The presence of CK in plasma is indicative of the tissue damage that may occur in powerful ischemic stress action to muscles, as for example in myocardial infarction.^11^ ADP to ATP conversion is a reversible reaction and Pcr therefore acts as a spatial and temporal buffer of ATP. Pcr is first synthesized in the liver, then transported via the bloodstream and finally stored in muscle cells and the brain. Therefore, Pcr plays a particularly important role in tissues that have high, fluctuating energy demands.^12^ Since irradiation impairs function of multiple organs^13^ (*i.e*. liver function) it is also possible to detect radiation effects by measuring liver enzyme levels aspartate aminotransferase (AST) and alanine aminotransferase (ALT).^14^ AST and ALT are parenchymal intracellular enzymes released into systemic circulation when there is hepatocellular injury and necrosis.

The metabolic changes of tissues and organs can be efficiently monitored by NMR spectroscopy methods^15^–^17^, in particular by phosphorous ^31^P and carbon ^13^C NMR spectroscopy methods. Phosphocreatine to adenosine triphosphate ratio (Pcr/ATP) as determined from corresponding spectral line peaks of ^31^P spectra is an appropriate index to follow energy metabolism.^18^,^19^ Until now few attempts have been made to detect effects of radiation by NMR spectroscopy. Ng *et al.* studied effects of gamma-irradiation on tumour cells by ^31^P NMR spectroscopy.^20^ They detected a dramatic decline in high-energy phosphates beginning one day after irradiation. Box *et al.* studied effects of radiation on degradation of glycine, a protein building block molecule, by ^13^C NMR spectroscopy.^21^ In addition to NMR spectroscopy methods, magnetic resonance (MR) imaging can be efficient in detecting effects of radiation as well. For example ^19^F MR imaging was employed to detect accumulation of perfluorooctylbromide in spleen.^22^ The accumulation was a consequence of macrophage dysfunction induced by irradiation.

The aim of this study was to examine relation between a received dose of ionizing irradiation and changes in metabolism that can be detected by ^31^P NMR spectroscopy of mice for potential determination of the received dose of radiation. The study is based on assumption that exposure of whole-body to high-dose radiation for only a short time period results in development of cell death, which presumably occurs due to uncoupling of oxidative phosphorylation and increased ion flux. This results in increased CK levels in serum and consequently decreased Pcr/ATP ratio due to homeostatic mechanisms responsible for energy supply. Assuming that the radiation affects multiple tissues, including liver, muscles and central nervous system, where is the major production and storage of Pcr, we expect the decrease of Pcr in irradiated mice and no significant change in the nonirradiated control group. If the assumption is right, the Pcr/ATP ratio could be used as a biosensor for the received dose of radiation provided that other mechanisms effecting Pcr/ATP ratio are excluded.

## Materials and methods

### Experimental animals and irradiation

In the experiments, C57Bl/6 mice raised at the Institute of Pathology (Medical Faculty, University of Ljubljana, Slovenia) were used. Mice were maintained at 21°C with natural day/night light cycle in a conventional animal colony. At the beginning of the experiments, mice, that were 16–20 weeks old, were subjected to an adaptation period of 7–10 days before experiments. Mice were divided equally between a control group (6 mice) that was not irradiated and a group that received 7 Gy of X-ray radiation (6 mice).

For X-ray irradiation a Darpac 2000 unit (Gulmay Medical Ltd, Shepperton, UK), operated at 220 kV, 10 mA, and with 0.55 mm Cu and 1.8 mm Al filtration was used. In experiments whole-body of mice was irradiated at a dose rate 2.2 Gy/min with single doses of 7 Gy. During whole body irradiation mice were anaesthetised with intraperitoneal injection of acepromazine (Promace, Fort Dodge Animal Health, Iowa, USA; 0.05 mg/mouse), ketamine hydrochloride (Bioketan, Vetoquinol, Paris, France; 2.5 mg/mouse) and xylazine hydrochloride (Rompun 2%, Bayer AG, Leverkusen, Germany; 0.25 mg/mouse).

Animal studies were carried out according to the guidelines of the Ministry of Agriculture, Forestry and Food of the Republic of Slovenia (permission No 34401-60/2007/8), and in compliance with the Guide for the Care and Use of Laboratory Animals (National Institutes of Health, Bethesda, MD). Protocol was approved by Veterinary administration of the Republic of Slovenia (34401-60/2007/8).

### ^31^P NMR Spectroscopy

Metabolic changes in both mice groups were measured using ^31^P NMR spectroscopy of the whole animal at the specific time of day for several days after the prime dose of radiation. The first measurement on irradiated mice was done immediately after the irradiation and then every other day for the next 14 days; no mice died within that period. The rest of the measurements on the control group were performed in five day intervals until three weeks after the experiment onset. NMR experiments were performed on a 2.35 T (100 MHz proton frequency) horizontal bore Oxford superconducting magnet (Oxford Instruments Ltd., UK) connected to a Tecmag Apollo spectrometer (TecMag, Huston TX, USA). ^31^P NMR signal was detected by a Bruker 4 cm double-tuned surface coil (Bruker, Ettlingen, Germany). For MRI, mice were fist anaesthetised using the same procedure as for X-ray irradiation. After that they were placed in the MRI magnet in the centre of the surface coil to focus signal acquisition on muscles and internal organs. A special care was taken in reproducibility of the animal placement (in the standard ventral position) relative to the surface coil. The coil was then tuned to proton signal for the purpose of magnet shimming which was done using the proton NMR signal. After the shimming was completed the coil was tuned to ^31^P and the ^31^P NMR signal acquisition started. The signal was acquired by the standard 1D acquisition sequence consisting of one 90° excitation pulse followed by the signal acquisition. The acquisition parameters were: acquisition size 4096 points, spectral width 10 kHz, acquisition time 200 ms, repetition time 2.2 s. The signal was averaged 1200 times so the total experiment time was 44 min. The spectra were reconstructed using 10 Hz exponential line broadening to decrease the signal noise. During the experiment the animals were coated with a layer of a cotton wool to prevent them dying from hypothermia.

### Measurement of creatine kinase and transaminases

The activities of creatine kinase (CK) and transaminases (AST and ALT) were measured in plasma of the irradiated mice with an automated biochemistry analyser RX Daytona (Randox, Crumlin, UK). Blood samples (200 μl) were collected from the orbital sinus by heparinised glass capillary before and after irradiation at different time points (10 min and 2, 4, 7, 9, 11 days). To prevent degradation of creatine kinase blood samples were centrifuged (3000 rpm, 10 min) in 10 min after dispossession. 100 μl of plasma samples were drawn in 1 ml tube and stored at −80°C until the analysis was performed.

### Statistical analysis

Measured Pcr/ATP ratios of the irradiated and the control mice group were analyzed for statistically significant difference by the two-tailed Student t-test (MS Excel 2007).

## Results

**FIGURE 1:**
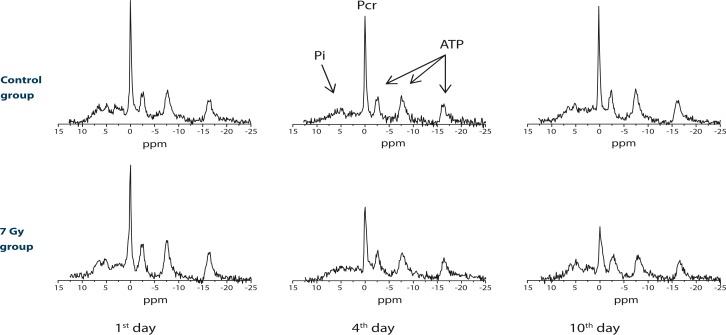
Typical ^31^P NMR spectra of X-ray irradiated mice at different times after the irradiation: immediately, after 4 days and after 10 days for the mice group that received 7 Gy of X-ray radiation (bottom row) and the control group that was not irradiated (top row).

Typical ^31^P NMR spectra of irradiated mice immediately after the irradiation, after 4 days and after 10 days are depicted in Figure 1 bottom row. For comparison, spectra of the control group acquired at identical time points are shown as well (Figure 1, top row). As expected, changes were significant only in spectra of irradiated mice, while in the control group, in which mice were not exposed to radiation, all spectra are alike and were changing with time significantly less. In Figure 1 it can clearly be seen that metabolic changes due to the irradiation are associated mainly with the reduced Pcr peak, while no significant difference was observed in heights of the average ATP peak and the inorganic phosphate peak (Pi). Therefore, the ratio between heights of the Pcr and the average ATP peak is a convenient measure for metabolic activity and can be used for following effects of radiation on energy metabolism.

**FIGURE 2:**
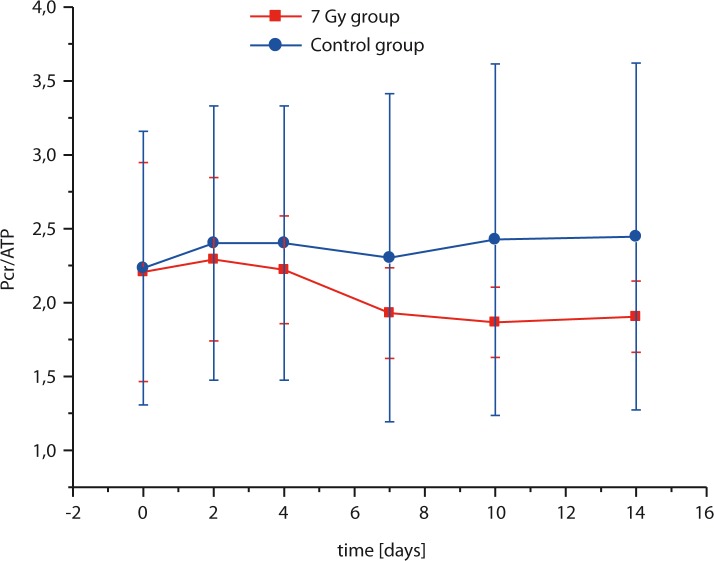
Pcr/ATP ratio as function of time after the irradiation for the mice group that received 7 Gy of X-ray radiation (squares) and the control mice group (circles). In the 7 Gy group the decrease of Pcr/ATP ratio is significant due to extensive radiation induced energy metabolism changes.

In Figure 2 dependence of the Pcr/ATP ratio as a function of time after the exposure to radiation for the mice group that received 7 Gy of X-ray radiation and the control mice group is depicted. In the control group, the Pcr/ATP ratio was practically constant all times, while the Pcr/ATP ratio in the irradiated group was initially identical to the Pcr/ATP ratio of the control group and then started decreasing until the animal death or partial recovery. The decrease was most significant within the first 7 days after the irradiation. After that time approximately half of the irradiated mice died and the other half never recovered completely, which can be seen by somewhat reduced Pcr/ATP ratio (reduction was approximately 20%) of the irradiated group compared to the same ratio of the control group for 7 or more days after the irradiation. Statistical analysis of the Pcr/ATP ratio by the paired t-test showed significant difference between the irradiated and the control mice group (*P* = 0.023).

**FIGURE 3:**
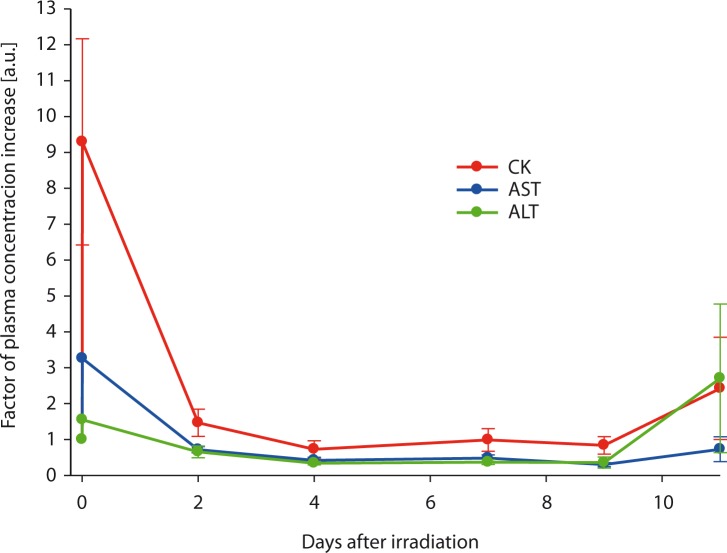
Relative creatine kinase (CK), aspartate aminotransferase (AST) and alanine aminotransferase (ALT) levels in plasma of mice that received 7 Gy of X-ray radiation as a function of time after the radiation exposure. Immediately after the exposure the CK level increase is almost tenfold while the increase of both transaminases (AST and ALT) was not as big, however, still significant.

Measurements of CK, AST and ALT levels in plasma of the irradiated mice as a function of time after radiation are shown in Figure 3. These measurements clearly indicate elevated CK levels immediately after the irradiation with almost tenfold increase of the CK level 10 min after the irradiation. The CK level then relatively fast returned to the normal level which was reached two days after the irradiation. Levels of both transaminases (ALT and AST) were elevated as well, however the increase was lower; the increase was approximately threefold for AST and 1.5-fold for ALT.

## Discussion

Results of this study clearly indicate an existing relation between changes of energy metabolism and effects of radiation. These were detected instantly by *in vivo* ^31^P NMR spectroscopy as well as with laboratory biochemical analysis of CK, AST and ALT levels in plasma, which was more time consuming. As expected, both methods were able to reveal metabolic changes associated with radiation effects. In ^31^P NMR the change was observed in the reduced Pcr/ATP ratio, while biochemical analysis of plasma revealed increased CK and less pronounced increase of AST and ALT levels. The spectra reveal the level of Pcr and ATP through the whole body (muscle and internal organs). The decrease in the Pcr/ATP ratio supports our assumption, that metabolism of Pcr in post-radiation time is elevated due to a higher Pcr level in serum, which represents a substrate of CK. Uncoupling oxidative phosphorylation after the irradiation due to cell death (or ascites cell mitochondria), creates a stressful environment with a lack of ATP.^23^ Therefore, its rapid replenishment could be achieved by resynthesis from Pcr and ADP, catalyzed by CK. Cell death results also in a decreased Pcr synthesis in liver and in a decreased Pcr storage in muscles due to rhabdomylosis-like effect of radiation, which can be seen in lover Pcr/ATP in irradiated group compared to the control group 20 or more days after the irradiation (Figure 2). The Pcr/ATP ratio decreased due to reduced intracellular creatine levels and therefore lower Pcr synthesis.^24^ It is expected that the decrease in the Pcr/ATP ratio is radiation-dose dependant, meaning that metabolism of Pcr would be affected even more if the radiation dose would be higher. In the irradiated mice group, in the first few days after the irradiation the ATP level was replenished by short-term mechanisms of the phosphate group transfer from Pcr to ADP. After that time, the ATP replenishment became due to severe radiation damage, which causes uncoupling of oxidative phosphorylation^25^ (*i.e*. liver damage) no longer possible thus resulting in the animal death with approximately 50% probability in 10 days after the irradiation.

The results of ^31^P NMR spectroscopy were confirmed biochemically by measuring CK levels in plasma. Parallel measurements of Pcr/ATP ratios and CK levels in plasma showed correlation between the ^31^P NMR spectroscopy and biochemical methods of assessment of effects of radiation; *i.e*., both methods showed initially increased CK ratios and decreased Pcr/ATP levels that returned to normal values during the recovery. Elevation of CK is an indication of acute muscle damage (*i.e* trauma, rhabdomyolysis, myocardial infarction, myositis etc.), which was in our study induce by X-ray radiation.^26^ In addition, increased levels of transaminases (AST and ALT) indicate a liver damage which supports our assumption that irradiation affected metabolism of multiple organs. While CK levels and levels of both transaminases returned to normal within two days after the irradiation, Pcr/ATP ratios remained decreased significantly longer (10 or more days). As long as CK levels in plasma are elevated ATP synthesis *de novo* is extremely slow which explains different time dynamics of the return for CK and Pcr levels to normal.

Limitations of our study are associated with the credibility of CK and Pcr/ATP peaks. For ^31^P NMR measurements surface coils were used. These have a sensitive region above the coil within a range approximately identical to the coil’s radius. Therefore a proper positioning of a mouse relative to the coil is important for acquisition of the NMR signal from always identical body parts. A failure to do so may result in significantly different spectra and consequently inaccurate determination of the Pcr/ATP ratio. In addition CK levels were measured non-selectively for all different CK isoenzymes. However, it is expected that most of CK originated mainly from skeletal muscles since the muscles represent the major storage for CK.

Due to the limited access to the X-ray radiation source the study was performed using only one dose of radiation (7 Gy, plus the control group that did not receive any radiation). Unfortunately this is not enough to determine a possible relation between the received dose and the Pcr/ATP ratio change. For that, similar experiments should be repeated for other intermediate doses (between 0 Gy and 7 Gy), which is our plan for future experiments. Although the chosen dose of 7 Gy is relatively high (50% mortality rate of mice), determination of such radiation exposure is important. At such high dose of radiation dose-dependent radiation-induced multi-organ involvement (RIMOI) and radiation-induced multi-organ failure (RIMOF) occur. Both RIMOI and RIMOF contribute to the clinical outcome and prognosis of radiation accident victims.^27^,^28^

## Conclusions

The NMR method for detection of radiation induced metabolic changes in living organisms was verified biochemically by analysing CK levels in plasma. The accuracy of the NMR method is inferior to standard dosimetry methods. However, its advantage is that the method is instantaneous and the radiation dose can still be determined even if the subject was not carrying a radiation detection device at the time of exposure. To determine possible relation between the Pcr/ATP ratio and the received dose of radiation prospective studies are still needed.
